# Supplementary material for: SERCA2 phosphorylation at serine 663 is a key regulator of Ca2+ homeostasis in heart diseases
Source: Nat Commun. 2023 Jun 8;14:3346. doi: 10.1038/s41467-023-39027-x (PMC10250397; doi:10.1038/s41467-023-39027-x)

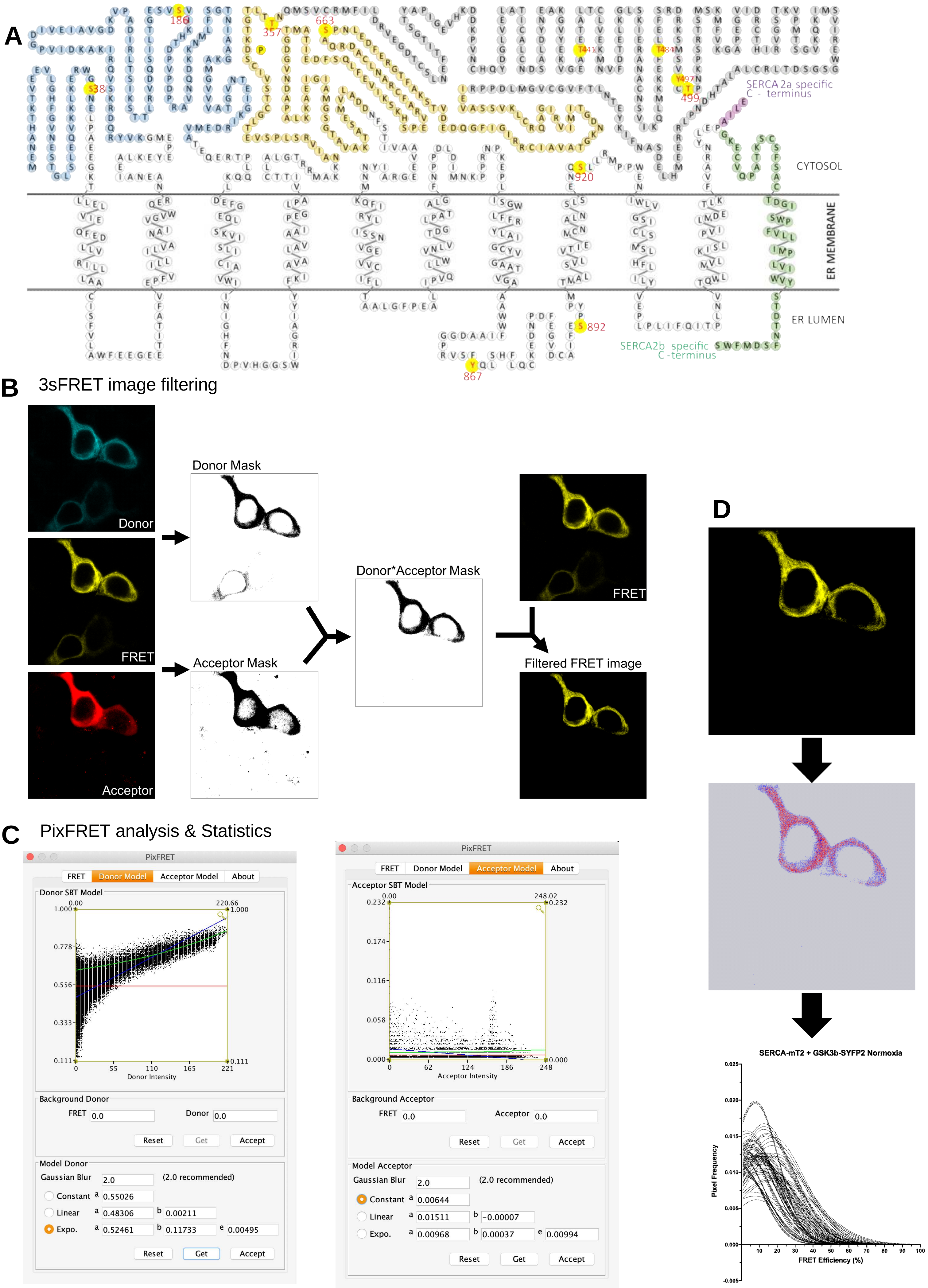
A
SERCA 2a specific
C - terminus
CYTOSOL
ER MEMBRANE
ER LUMEN
SERCA2b specific
C -terminus
186
357
663
S38
T441
T484
Y497
499
N920
892
867
B
3sFRET image filtering
Donor
FRET
Acceptor
Donor Mask
Acceptor Mask
Donor*Acceptor Mask
FRET
Filtered FRET image
C
PixFRET analysis & Statistics
PixFRET
FRET
Donor Model
Acceptor Model
About
Donor SBT Model
Donor Intensity
Background Donor
Model Donor
Gaussian Blur
(2.0 recommended)
Constant
Linear
Expo.
Reset
Get
Accept
Acceptor SBT Model
Acceptor Intensity
Background Acceptor
Model Acceptor
D
SERCA-mT2 + GSK3b-SYFP2 Normoxia
Pixel Frequency
FRET Efficiency (%)

Supplemental Figure 1. Putative phosphorylation target sites of SERCA2 for the GSK3β kinase. (A) Mapping of the eleven putative GSK3β target sites on SERCA2 sequence (yellow), with activation (blue), phosphorylation (brown) and nucleotide-binding (green) domains of SERCA2. **(B)** 3sFRET image filtering. **(C)** PixFRET analysis and statistics. **(D)** FRET efficiency maps

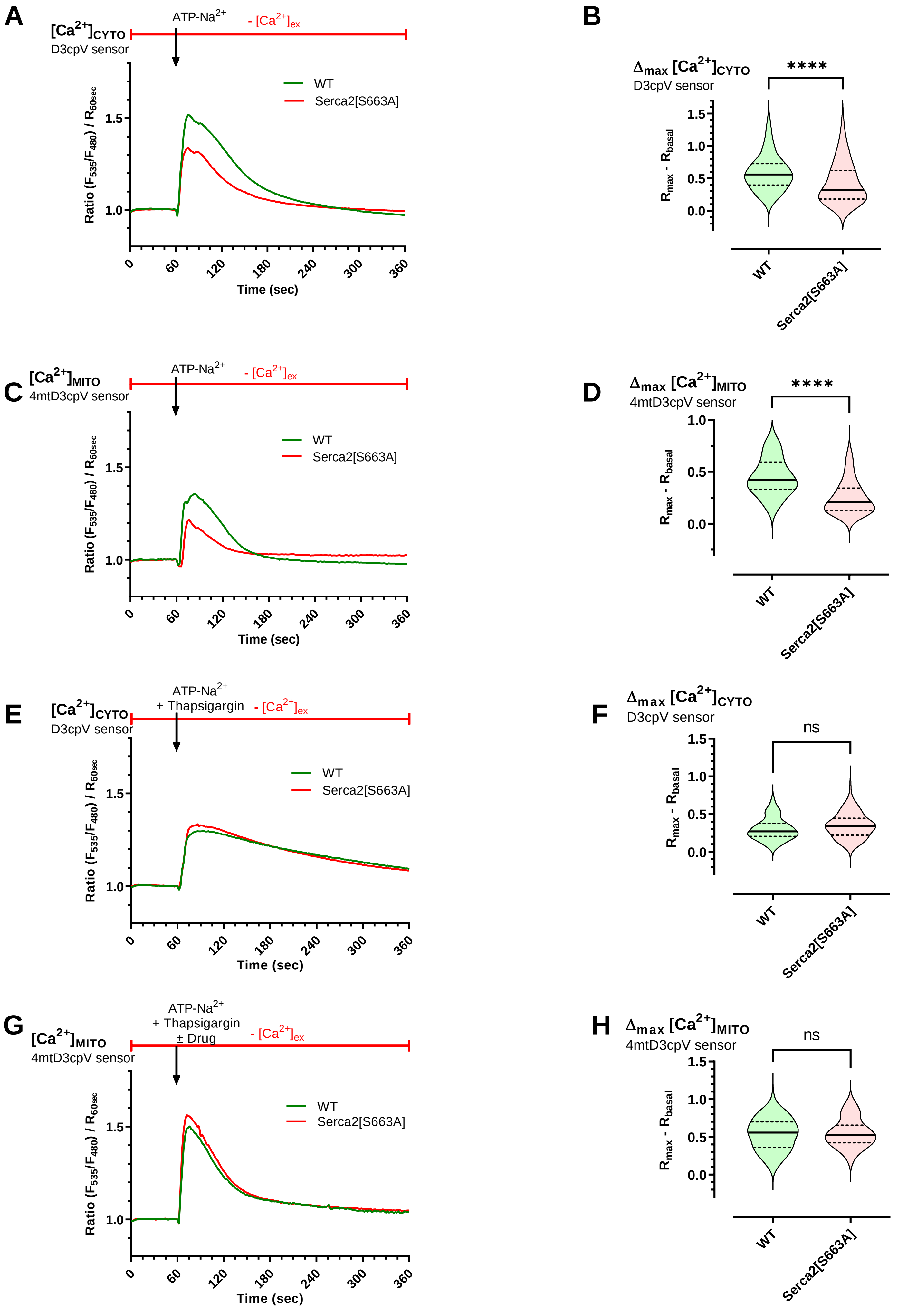

A
$[Ca^{2+}]_{CYTO}$
D3cpV sensor
ATP-Na^{2+}
- $[Ca^{2+}]_{ex}$
WT
Serca2[S663A]
Ratio (F_{535}/F_{480}) / R_{60sec}
Time (sec)
B
Δ_{max} $[Ca^{2+}]_{CYTO}$
D3cpV sensor
****
R_{max} - R_{basal}
WT
Serca2[S663A]
C
$[Ca^{2+}]_{MITO}$
4mtD3cpV sensor
ATP-Na^{2+}
- $[Ca^{2+}]_{ex}$
WT
Serca2[S663A]
Ratio (F_{535}/F_{480}) / R_{60sec}
Time (sec)
D
Δ_{max} $[Ca^{2+}]_{MITO}$
4mtD3cpV sensor
****
R_{max} - R_{basal}
WT
Serca2[S663A]
E
$[Ca^{2+}]_{CYTO}$
D3cpV sensor
ATP-Na^{2+}
+ Thapsigargin
- $[Ca^{2+}]_{ex}$
WT
Serca2[S663A]
Ratio (F_{535}/F_{480}) / R_{60sec}
Time (sec)
F
Δ_{max} $[Ca^{2+}]_{CYTO}$
D3cpV sensor
ns
R_{max} - R_{basal}
WT
Serca2[S663A]
G
$[Ca^{2+}]_{MITO}$
4mtD3cpV sensor
ATP-Na^{2+}
+ Thapsigargin
± Drug
- $[Ca^{2+}]_{ex}$
WT
Serca2[S663A]
Ratio (F_{535}/F_{480}) / R_{60sec}
Time (sec)
H
Δ_{max} $[Ca^{2+}]_{MITO}$
4mtD3cpV sensor
ns
R_{max} - R_{basal}
WT
Serca2[S663A]

Supplemental Figure 2. Influence of phosphoresistance of SERCA2 at S663 on IP3R-induced calcium response. Representative curves of normalized $[Ca^{2+}]_{CYTO}$ (cytosol) and $[Ca^{2+}]_{MITO}$ (mitochondria) ratio signals over time measured with D3cpV and 4mtD3cpV probes respectively, in HEK293-T wild-type (WT) (green curve) and SERCA2[S663A] phosphoresistant mutant (red curve) cells in Ca^{2+} free and 1mM EGTA-containing buffer for one minute prior to add 100µM ATP-Na_2 with **(E,G)** or without **(A,C)** 1µM thapsigargin. **(B)** Violin plot of normalized $[Ca^{2+}]_{CYTO}$ maximal increase after ATP-Na^{2+} stimulation in WT (N=141 cells on 7 independent days) and SERCA2[S663A] phosphoresistant mutant (N=197 cells on 10 independent days). Median with interquartile range is shown and two-tailed unpaired t test with Welch's correction was used to assess significance (****$p < 0.0001$). **(D)** Violin plot of normalized $[Ca^{2+}]_{MITO}$ maximal increase after ATP-Na^{2+} stimulation in WT (N=141 cells on 7 independent days) and SERCA2[S663A] phosphoresistant mutant (N=197 cells on 10 independent days). Median with interquartile range is shown and two-tailed unpaired t test with Welch's correction was used to assess significance (****$p < 0.0001$). **(F)** Violin plot of normalized $[Ca^{2+}]_{CYTO}$ maximal increase after ATP-Na^{2+} stimulation in presence of thapsigargin in WT (N=90 cells on 7 independent days) and SERCA2[S663A] phosphoresistant mutant (N=129 cells on 10 independent days). Median with interquartile range is shown and two-tailed unpaired t test with Welch's correction was used to assess significance ns $p \geq 0.05$). **(H)** Violin plot of normalized $[Ca^{2+}]_{MITO}$ maximal increase after ATP-Na^{2+} stimulation in presence of thapsigargin in WT (N=83 cells on 7 independent days) and SERCA2[S663A] phosphoresistant mutant (N=72 cells on 7 independent days). Median with interquartile range is shown and two-tailed unpaired t test with Welch's correction was used to assess significance (ns $p \geq 0.05$).

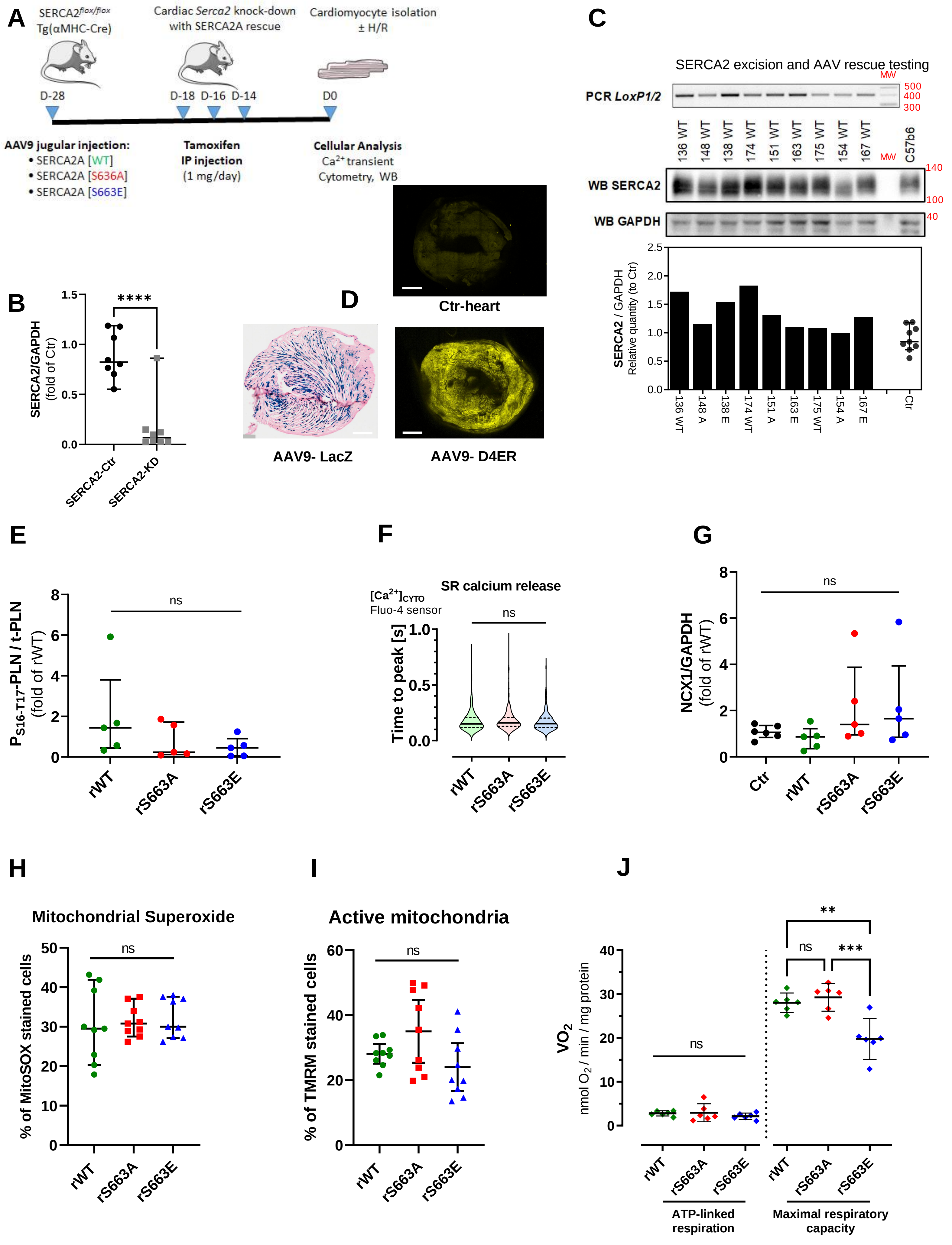

A
SERCA2flox/flox Tg(αMHC-Cre)
Cardiac Serca2 knock-down with SERCA2A rescue
Cardiomyocyte isolation ± H/R
D-28
D-18 D-16 D-14
D0
AAV9 jugular injection:
• SERCA2A [WT]
• SERCA2A [S636A]
• SERCA2A [S663E]
Tamoxifen IP injection (1 mg/day)
Cellular Analysis
Ca2+ transient
Cytometry, WB
B
SERCA2/GAPDH (fold of Ctr)
****
SERCA2-Ctr
SERCA2-KD
C
SERCA2 excision and AAV rescue testing
PCR LoxP1/2
WB SERCA2
WB GAPDH
MW
136 WT 148 WT 138 WT 174 WT 151 WT 163 WT 175 WT 154 WT 167 WT C57b6
SERCA2 / GAPDH Relative quantity (to Ctr)
136 WT 148 A 138 E 174 WT 151 A 163 E 175 WT 154 A 167 E Ctr
D
Ctr-heart
AAV9- LacZ
AAV9- D4ER
E
PS16-T17-PLN / t-PLN (fold of rWT)
ns
rWT rS663A rS663E
F
SR calcium release
[Ca2+]CYTO
Fluo-4 sensor
Time to peak [s]
ns
rWT rS663A rS663E
G
NCX1/GAPDH (fold of rWT)
ns
Ctr rWT rS663A rS663E
H
Mitochondrial Superoxide
% of MitoSOX stained cells
ns
rWT rS663A rS663E
I
Active mitochondria
% of TMRM stained cells
ns
rWT rS663A rS663E
J
VO2 nmol O2 / min / mg protein
ns
**
***
rWT rS663A rS663E
ATP-linked respiration
Maximal respiratory capacity

Supplemental Figure 3. Phenotyping of phosphoresistant and phosphomimetic SERCA2 at S663 in adult cardiomyocytes. (A) *In vivo* experimental design for *in vitro* experiments on isolated cardiomyocytes or heart transverse section rescued with SERCA2 -WT, -S663A or -S663E mutant. **(B)** Dot plot of SERCA2 expression in C57Bl6J mice (Ctr) (N=8) and knockdown (KD) mice (N= 8), normalized as fold of Ctr. Median with interquartile interval, on 2 independent days, is shown and two-tailed unpaired t test with Welch's correction was used to assess significance (****p<0.0001). **(C)** Top panel, *LoxP1/2* endpoint PCR genotyping for endogenous *Serca2* excision testing after Tamoxifen treatment (n=3/group). Lower panel, representative Western blotting for phenotyping of SERCA2 rescue by AAVs. (Numbers correspond to individual mouse identification numbers). Median with interquartile interval is shown for Ctr (N=9). **(D)** Confocal images of heart slices acquired with objective 10X. Mosaic Z-stack images of 65µm thickness were acquired. The images are representing the maximum intensity projection of 5 Z-stack images. Confocal images of Ctr heart (top-right panel) and AAV9-D4ER heart (bottom-right panel). For AAV9-LacZ (bottom-left panel), 25µm-cryosections were stained with 5-bromo-4-chloro-3-indolyl-β-D-galactopyranoside to reveal β galactosidase activity and images were manually scanned using Olympus microscope (BX63 microscope, Japan) at objective 10x after eosin counterstaining). Scale bar represents 1000µm **(E)** Dot plot of phosphorylation of phospholamban at serine 16 and threonine 17, normalized with total phospholamban from rWT (N=5), rS663A (N=5) and rS663E (N=5) cardiomyocyte isolations. Median with interquartile range, on 3 independent days, is shown and one-way ANOVA followed by Tukey's multiple comparisons test was used to assess significance (ns p≥0.05). **(F)** Violin plot of the time to peak of the $[Ca^{2+}]_{CYTO}$ (cytosol) after caffeine stimulation of rWT (N=180), rS663A (N=251) and rS663E (N=175) cardiomyocytes . Median with interquartile range, on 7 independent days, is shown and one-way ANOVA was used to assess significance (ns p≥0.05). **(G)** Dot plot of NCX1 normalized by GAPDH of Ctr (N=6), rWT (N=5), rS663A (N=5) and rS663E (N=5) cardiomyocytes isolation. Median expressed as fold of rWT with interquartile range, on 3 independent experiment days, is shown and one-way ANOVA followed by Tukey's multiple comparisons test was used to assess significance (ns p≥0.05). **(H)** Flow cytometry analysis of oxidative stress evaluated with mitochondrial superoxide (MitoSOX) staining from rWT (N=9), rS663A (N=9) and rS663E (N=9) cardiomyocyte isolations. Mean with a 95% confidence interval, on 3 independent experimental days, is shown and one-way ANOVA followed by Tukey's multiple comparisons test was used to assess significance (ns p>0.05). **(I)** Flow cytometry analysis of active mitochondria by tetramethylrhodamine methyl ester (TMRM) staining from rWT (N=9), rS663A (N=9) and rS663E (N=9) cardiomyocyte isolations. Mean with a 95% confidence interval is shown, on 3 independent days, and one-way ANOVA followed by Tukey's multiple comparisons test was used to assess significance (ns p>0.05). **(J)** Dot plot of the OXPHOS of cardiomyocytes isolated from 6 mice in each group. ATP-linked respiration was derived from the difference between basal oxygen consumption rate and following oligomycin addition. Maximal respiratory capacity was measured by FCCP stimulation to reach the highest oxygen consumption rate. Mean with a 95% confidence interval is shown and Kruskal-Wallis test followed by Dunn's multiple comparisons test was used to assess significance (ns p=0.7991 rWT vs rS663A, **p=0.0018, ***p=0.005).

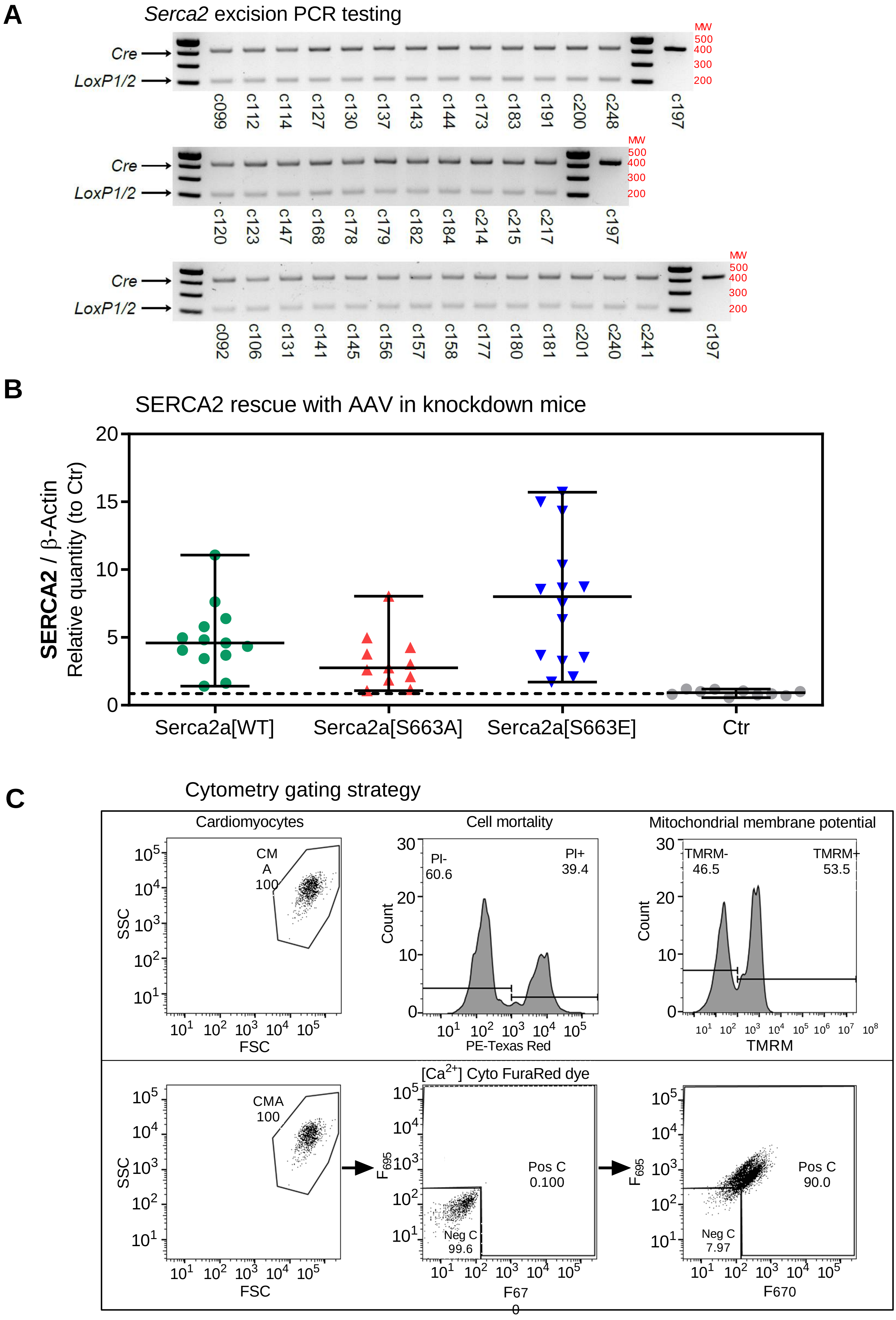
A
Serca2 excision PCR testing
Cre
LoxP1/2
MW
500
400
300
200
c099 c112 c114 c127 c130 c137 c143 c144 c173 c183 c191 c200 c248 c197
c120 c123 c147 c168 c178 c179 c182 c184 c214 c215 c217 c197
c092 c106 c131 c141 c145 c156 c157 c158 c177 c180 c181 c201 c240 c241 c197
B
SERCA2 rescue with AAV in knockdown mice
SERCA2 / β-Actin
Relative quantity (to Ctr)
Serca2a[WT]
Serca2a[S663A]
Serca2a[S663E]
Ctr
C
Cytometry gating strategy
Cardiomyocytes
CM A 100
SSC
FSC
Cell mortality
PI- 60.6
PI+ 39.4
Count
PE-Texas Red
Mitochondrial membrane potential
TMRM- 46.5
TMRM+ 53.5
TMRM
[Ca2+] Cyto FuraRed dye
CMA 100
F695
Pos C 0.100
Neg C 99.6
F670
Pos C 90.0
Neg C 7.97

Supplemental Figure 4. Properties of *Serca2*-knockdown mice used for *in vivo* experiments. (A) Endpoint multiplex PCR genotyping *Cre* and *LoxP1/2* to detect Cre recombinase presence and endogenous *Serca2* excision after Tamoxifen treatment, respectively. The c197 *Serca2*$^{cre/lox}$ transgenic mouse was used as a control without Tamoxifen treatment. **(B)** Dot plot of western blotting phenotyping for SERCA2 rescue strategy by AAVs in Serca2[WT] (N=13), Serca2[S663A] (N=11), Serca2[S663E] (N=14) rescued and C57BL/6 control mice (Ctr) (N=10). Median with range is shown. **(C)** Cytometry gating strategy for cell mortality, mitochondrial membrane potential and resting of cytosol Ca^{2+} for cardiomyocytes isolated from AAR after the *in vivo* ischemia-reperfusion insult.

Supplementary Source data:

WB Supplemental Figure 3C

2020-08-13 15hr 41min_Exposure_8.5sec+Colorimetric

SERCA2b (140kDa) ▶
SERCA2a (114kDa) ▶

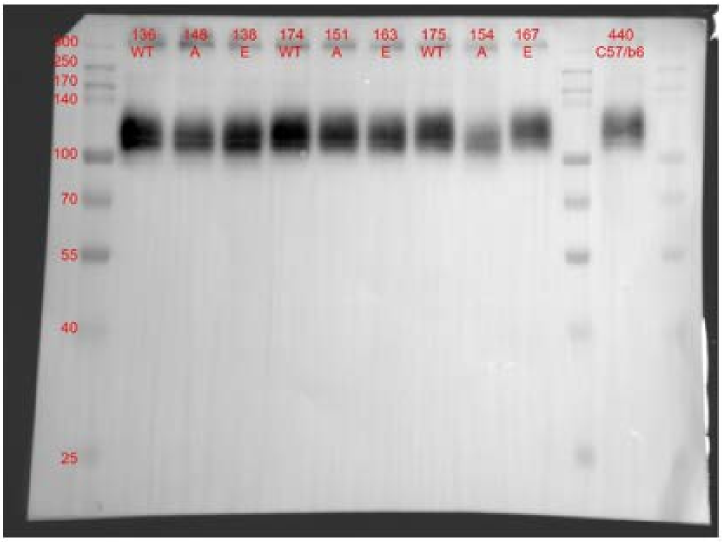

GAPDH (37kDa) ▶

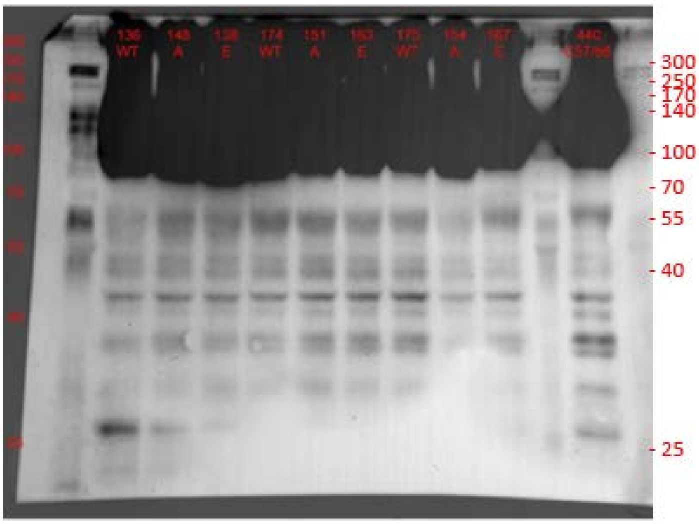

<u>**Gels Supplemental Figure 4A**</u>

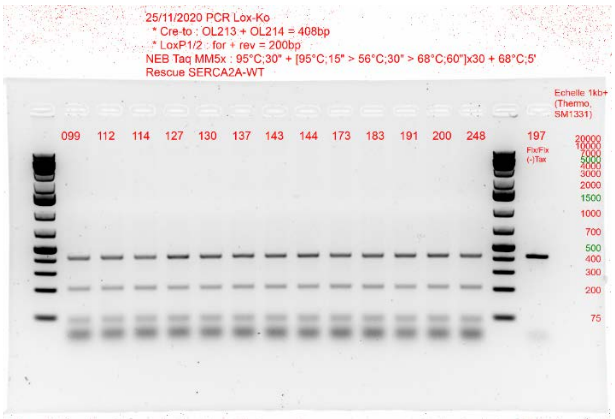

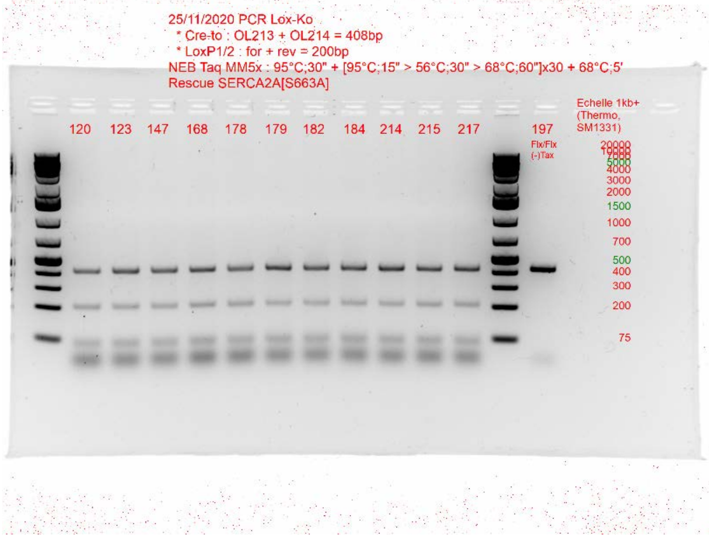

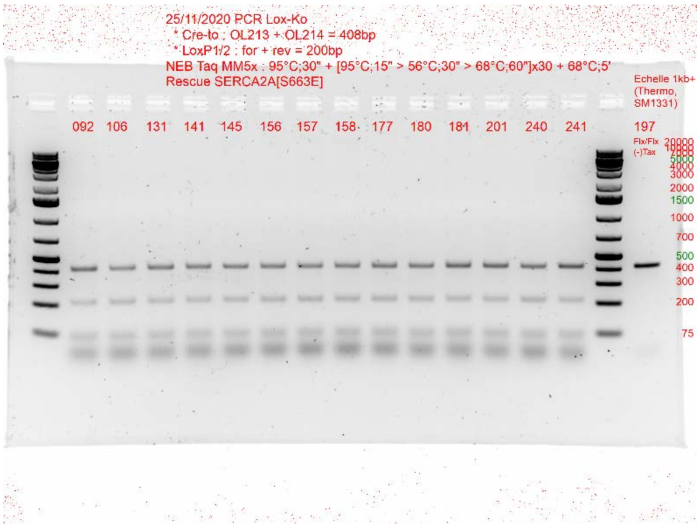

WB Supplemental Figure 4B

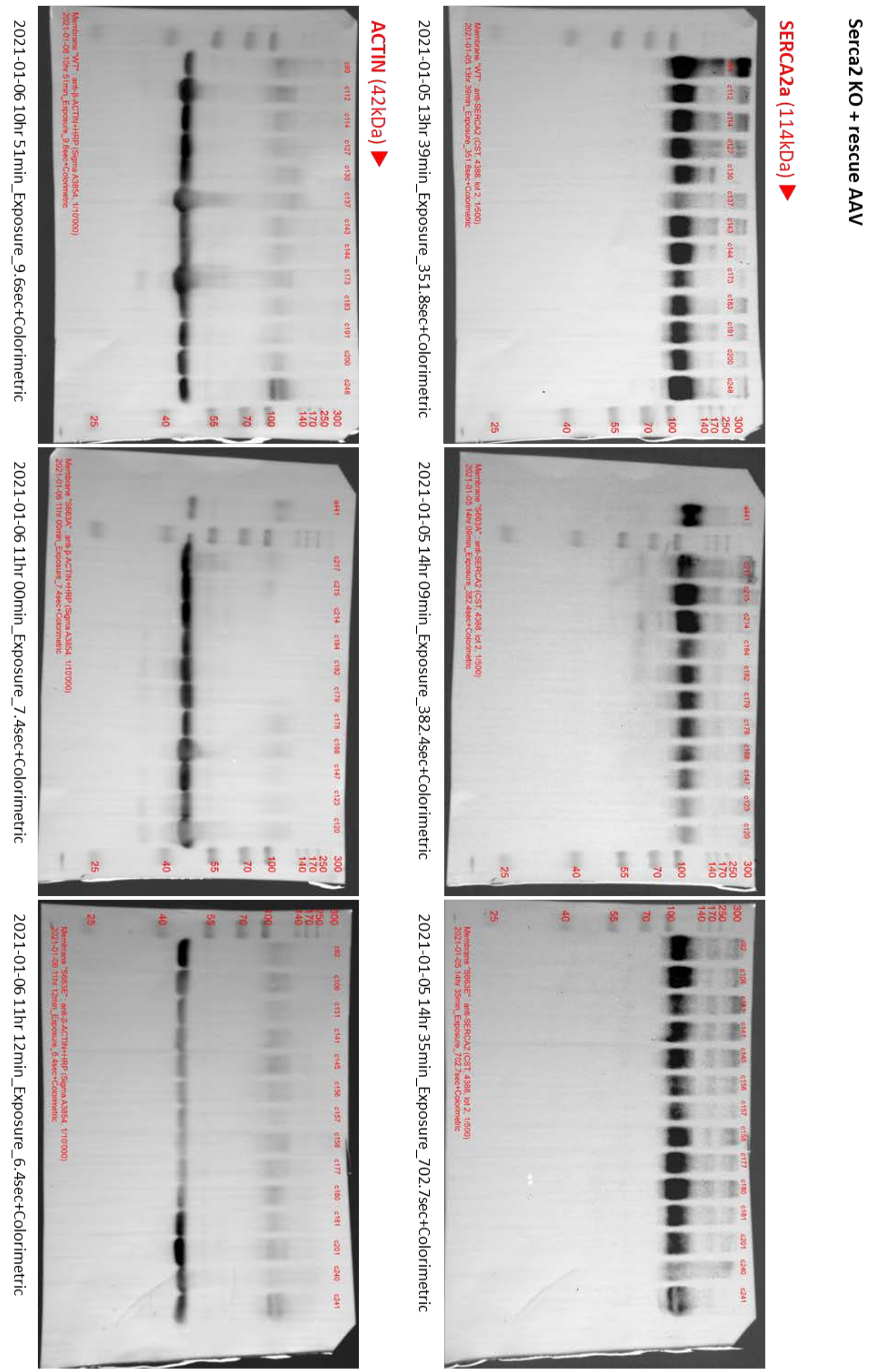

Supplement: Supplementary file 1 — Supplementary Information [file 41467_2023_39027_MOESM1_ESM.pdf]
