# Supplementary material for: SERCA2 phosphorylation at serine 663 is a key regulator of Ca2+ homeostasis in heart diseases
Source: Nat Commun. 2023 Jun 8;14:3346. doi: 10.1038/s41467-023-39027-x (PMC10250397; doi:10.1038/s41467-023-39027-x)
Supplement: Supplementary file 3 — Reporting Summary [file 41467_2023_39027_MOESM3_ESM.pdf]

nature portfolio

Corresponding author(s): Ludovic GOMEZ

Last updated by author(s): May 22, 2023

Reporting Summary

Nature Portfolio wishes to improve the reproducibility of the work that we publish. This form provides structure for consistency and transparency in reporting. For further information on Nature Portfolio policies, see our Editorial Policies and the Editorial Policy Checklist.

Statistics

For all statistical analyses, confirm that the following items are present in the figure legend, table legend, main text, or Methods section.

n/a	Confirmed	
[ ]	[x]	The exact sample size (*n*) for each experimental group/condition, given as a discrete number and unit of measurement
[ ]	[x]	A statement on whether measurements were taken from distinct samples or whether the same sample was measured repeatedly
[ ]	[x]	The statistical test(s) used AND whether they are one- or two-sided<br>*Only common tests should be described solely by name; describe more complex techniques in the Methods section.*
[ ]	[x]	A description of all covariates tested
[ ]	[x]	A description of any assumptions or corrections, such as tests of normality and adjustment for multiple comparisons
[ ]	[x]	A full description of the statistical parameters including central tendency (e.g. means) or other basic estimates (e.g. regression coefficient) AND variation (e.g. standard deviation) or associated estimates of uncertainty (e.g. confidence intervals)
[ ]	[x]	For null hypothesis testing, the test statistic (e.g. *F*, *t*, *r*) with confidence intervals, effect sizes, degrees of freedom and *P* value noted<br>*Give P values as exact values whenever suitable.*
[x]	[ ]	For Bayesian analysis, information on the choice of priors and Markov chain Monte Carlo settings
[x]	[ ]	For hierarchical and complex designs, identification of the appropriate level for tests and full reporting of outcomes
[x]	[ ]	Estimates of effect sizes (e.g. Cohen's *d*, Pearson's *r*), indicating how they were calculated

Our web collection on statistics for biologists contains articles on many of the points above.

Software and code

Policy information about availability of computer code

Data collection	The putative phosphorylation sites were predicted using NetPhos 3.1 and PhosphoSitePlus public databases.
Data analysis	-The fluorescent ratios were analyzed with MetaFluor 6.3 (Universal Imaging).<br>-3sFRET images were preprocessed by a home-made macro in ImageJ (Fiji 2.9.0) environment. Pixel distribution histograms of these FRET efficiency maps were extracted and fitted with a robust Gaussian regression (Prism 9.4.1, GraphPad Software).<br>-ImageJ (Fiji 2.9.0) software (National Institutes of Health) was used to quantify PLA experiments.<br>-Flow cytometry experiments were analysed using FACSDiva 8.0.1 software (Becton-Dickinson).<br>-Sarcomere shortening was analyzed using IonWizard Ionoptix 7.5.1<br>-Oxygen consumption was evaluated by the Oroboros DatLab4 software.<br>-Caffeine response paramaters were analyzed with OriginPRO (OriginLab9.0.0.87).<br>-Statistical analyses were conducted with Excel (Microsoft 2016), Prism (GraphPad 9.4.1), OriginPro (OriginLab9.0.0.87) and R (R Core Team 2020) software.<br>-Infarct size measurement with SigmaScan Pro 5.0.0<br>-Blotting were analysed with ImageLab 5.2.1<br>-Figures were partly generated using Servier Medical Art, provided by Servier, licensed under a Creative Commons Attribution 3.0 unported license.

For manuscripts utilizing custom algorithms or software that are central to the research but not yet described in published literature, software must be made available to editors and reviewers. We strongly encourage code deposition in a community repository (e.g. GitHub). See the Nature Portfolio guidelines for submitting code & software for further information.

Data

Policy information about availability of data

All manuscripts must include a data availability statement. This statement should provide the following information, where applicable:

- Accession codes, unique identifiers, or web links for publicly available datasets
- A description of any restrictions on data availability
- For clinical datasets or third party data, please ensure that the statement adheres to our policy

The data supporting this article and other findings are available from the corresponding authors upon request. Source data are provided with this paper

Human research participants

Policy information about studies involving human research participants and Sex and Gender in Research.

Reporting on sex and gender	According to the written consent, no sex and gender was collected in this preclinical research study.<br>Failing heart tissues were processed at the Louis Pradel hospital, Hospices Civils de Lyon and obtained with informed consent from explanted heart of patient admitted for heart transplantation (with severe reduction of ejection fraction (DCM, ICM)).<br>Non-failing heart samples and associated data were obtained with informed consent from Tissu-Tumorotheque Cardiobiotec (CRB-HCL, Hospices Civils de Lyon Biobank, BB-0033-00046 after Research Ethic Committee approval AC-2019-3464 and AC-2020-3918.
Population characteristics	inclusion:<br>- All patients that are 18 years of age or older<br>- Male/Female<br>-Failing heart tissues were collected from explanted heart of patient admitted for heart transplantation (severe reduction of EF with CMD or CMI indifferently).<br>- Non-failing heart samples were obtained from transplanted patients with no sign of cardiac graft rejection grade (grade 0)
Recruitment	Recruitment of failing heart patients was done during pre-assessment clinics or on the day of surgery.<br>Non-failing heart samples were obtained from Tissu-Tumorotheque Cardiobiotec of Lyon.
Ethics oversight	Heart samples and associated data were obtained with informed consent from Tissu-Tumorotheque Cardiobiotec (CRB-HCL, Hospices Civils de Lyon Biobank, BB-0033-00046 after Research Ethic Committee approval AC-2019-3464 and AC-2020-3918.

Note that full information on the approval of the study protocol must also be provided in the manuscript.

Field-specific reporting

Please select the one below that is the best fit for your research. If you are not sure, read the appropriate sections before making your selection.

☒ Life sciences ☐ Behavioural & social sciences ☐ Ecological, evolutionary & environmental sciences

For a reference copy of the document with all sections, see nature.com/documents/nr-reporting-summary-flat.pdf

Life sciences study design

All studies must disclose on these points even when the disclosure is negative.

Sample size	For imaging, 180-260 cells were acquired to obtain sufficient statistic power, in accordance with historical lab data (i.e. doi: 10.1007/s00395-020-00835-7; doi: 10.1038/s41419-020-02864-5 and doi: 10.1038/cdd.2015.101)<br>For mass spectrometry, n=10 was calculated for a 1.375 effect size with a variability of 8%, alpha 5% and beta 80% for mice experiments; and n=5 was calculated for a 2.33 effect size with a variability of 12%, alpha 5% and beta 80% for human experiments.<br>For cytometry, the total cell number threshold was set at 1,000 events for ACM and 10,000 events/acquisition for HEK, with n=10-12 per group on 4 different experimental days, as previously described (doi: 10.1152/ajpcell.00393.2019.)<br>For in vivo, n=10-14 was used to define any possible cardioprotection effect in accordance with historical lab data based a 34% effect with a variability of 10%, alpha 5% and beta 80% (doi: 10.1038/cdd.2015.101 ; doi: 10.1038/cdd.2015.101. ; doi: 10.1038/cdd.2015.118).
Data exclusions	all data were included in this study
Replication	For experiments involving protein quantification and flow cytometry, n=3 was chosen as the minimal replicate number. We determined this to be sufficient owing to internal control.<br>For Ca2+ measurements experiments were done on 2-11 distinct experimental days (6 different experimental days in average).<br>For mass spectrometry and in vivo experiments, measures were not replicated due to the scarcity of the sample and the power of the analysis.<br>For protein interaction data were done on 3-4 different experimental days.

	For electrophysiology, data were done on 3-4 different experimental days.
Randomization	in vivo and invitro experimental groups were randomized with a balance between studied groups (WT, phosphoresisant & phosphomimetic) before experimentation days .
Blinding	All investigators were blinded for in vitro and in vivo data collections and for analysis.

Reporting for specific materials, systems and methods

We require information from authors about some types of materials, experimental systems and methods used in many studies. Here, indicate whether each material, system or method listed is relevant to your study. If you are not sure if a list item applies to your research, read the appropriate section before selecting a response.

Materials & experimental systems

n/a	Involved in the study
☐	☒ Antibodies
☐	☒ Eukaryotic cell lines
☒	☐ Palaeontology and archaeology
☐	☒ Animals and other organisms
☒	☐ Clinical data
☒	☐ Dual use research of concern

Methods

n/a	Involved in the study
☒	☐ ChIP-seq
☐	☒ Flow cytometry
☒	☐ MRI-based neuroimaging

Antibodies

Antibodies used	rabbit anti-SERCA2 (Cell Signaling, 4388; 1/500); mouse anti-GSK3β (Abcam, ab-93926; 1/4000); mouse anti-GSK3β-phospho S9 (Abcam, ab-54537; 1/1000); rabbit anti-GSK3β-phospho Y216 (Abcam, ab-75745; 1/1000); mouse anti-PLN (Abcam, ab-2865; 1/4000); rabbit anti pS16-T17-PLN (Cell signaling 8496; 1/4000); rabbit anti-GAPDH (Santa Cruz, sc-25778; 1/2000); anti-βactin (Sigma, A3854; 1/10000); rabbit anti NCX1 (Abcam ab177952; 1/1000); rabbit anti pS727 STAT3 (Adcam ab86430); anti mouse STAT3 (Santacruz, sc8019; 1/1000); anti rabbit p44/42ERK (Cell signalling, cs9102; 1/1000); anti mouse total ERK1-2 (MAB1576; 1/1000). Blots were incubated with horseradish peroxidase (HRP)-coupled sheep anti-mouse IgG (GE Healthcare, NA931VS; 1/10000) and (HRP)-coupled goat anti-rabbit IgG (GE Healthcare, NA934VS; 1/10000), and developed with Clarity Western ECL Substrate (BioRad, 1705060).
Validation	Antibody experiments were done accordingly to the manufacturer (cf. Ab references) or according to previous published data of our lab. No additional validation was done on antibody experiment.

Eukaryotic cell lines

Policy information about cell lines and Sex and Gender in Research

Cell line source(s)	HEK293-T were obtained from ATCC (CRL-3216). hiPS control cell line (named AG08C5) was generated from primary fibroblasts (Coriell, ref. AG08498). MEF-T cells were isolated from 11.5 to 13.5-days post coïtum mouse embryos from genetically engineered mouse strains Serca2flox/flox. MEFs were immortalized thanks to a large T-antigen plasmid (Addgene #18922), then they were selected with 1 µg/mL Puromycin (Sigma, P8833) and cloned to obtain a MEF-T cell line.
Authentication	None of the cell lines have been authenticated.
Mycoplasma contamination	Cells were tested negative for mycoplasma contamination
Commonly misidentified lines (See ICLAC register)	No commonly misidentified cell lines were used.

Animals and other research organisms

Policy information about studies involving animals; ARRIVE guidelines recommended for reporting animal research, and Sex and Gender in Research

Laboratory animals	All experiments were performed on a parity of male and female mice of 8-12 weeks of age. The Serca2flox/floxTg(αMHC-MerCreMer) and Serca2flox/flox transgenic mice were kindly provided by Experimental Medical Research at Oslo University Hospital Ullevål (OUH-U) on a C57Bl/6 background.<br>Animals were housed in stable groups of four in individually ventilated cages (Nextgen - Allentown, USA – conventional animal facility) with standard nesting materials (cotton, tunnel) and ad libitum access to filtered water and standard diet (2018 global rodent diet, Envigo, France). Room temperature (housing and experiment) were maintained at 22°C ± 2°C and light cycle were at 12:12.
Wild animals	The study did not involve wild animals.

Reporting on sex	All experiments were performed on a parity of male and female mice
Field-collected samples	Animals were housed in stable groups of four in individually ventilated cages (Nextgen - Allentown, USA – conventional animal facility) with standard nesting materials (cotton, tunnel) and ad libitum access to filtered water and standard diet (2018 global rodent diet, Envigo, France). Room temperature (housing and experiment) were maintained at 22°C ± 2°C and light cycle were at 12:12.
Ethics oversight	All animal experiments were conducted in accordance with the Claude Bernard University of Lyon ethics committee CE2A-55 (approval no.: APAFIS#19896-201903212127912v2)

Note that full information on the approval of the study protocol must also be provided in the manuscript.

Flow Cytometry

Plots

Confirm that:

- [x] The axis labels state the marker and fluorochrome used (e.g. CD4-FITC).
- [x] The axis scales are clearly visible. Include numbers along axes only for bottom left plot of group (a 'group' is an analysis of identical markers).
- [x] All plots are contour plots with outliers or pseudocolor plots.
- [x] A numerical value for number of cells or percentage (with statistics) is provided.

Methodology

Sample preparation	Live ACM were labelled with 5 µM MitoSOX red reagent (Invitrogen, M36008) for detecting mitochondrial reactive oxygen species (ROS) and 20 nM MitoProbe Tetramethylrhodamine (Invitrogen, M20036) for determining mitochondrial membrane potential.
Instrument	Percentage of stained cells was measured using the 561 nm laser on a LSRFortessa X-20 (Becton-Dickinson)
Software	percentage of stained cells was analysed using FACSDiva 8.0.1 software (Becton-Dickinson).
Cell population abundance	The total cell number threshold for flow cytometry was set at 1,000 events for cardiomyocytes and 10,000 events for HEK cells.
Gating strategy	Strategy gating : a threshold of forward scatter (5000) was applied to eliminate debris and non-myocytes cells (Paccalet et al, Am J Physiol Cell Physiol. 2020 Feb 1;318(2):C439-C447. doi: 10.1152/ajpcell.00393.2019.)<br>A figure exemplifying the gating strategy was added to the supplemental data file.

- [x] Tick this box to confirm that a figure exemplifying the gating strategy is provided in the Supplementary Information.
